# Supplementary material for: Extraction, Characterization, and Platelet Inhibitory Effects of Two Polysaccharides from the Cs-4 Fungus
Source: Int J Mol Sci. 2022 Oct 20;23(20):12608. doi: 10.3390/ijms232012608 (PMC9604242; doi:10.3390/ijms232012608)
Supplement: Supplementary file 1 [file ijms-23-12608-s001.zip › ijms-1939852-supplementary.pdf]

Supplementary data

Table S1 Linkage analysis of EPSp and IPSp

Linkages	Methylated sugars	Relative molar ratio (%)
EPSp		
t-Ara(f)	1,4-di-O-acetyl-2,3,5-tri-O-methyl arabinitol	5.236
t-Fuc(p)	1,5-di-O-acetyl-6-deoxy-2,3,4-tri-O-methyl fucitol	0.900
t-Man(p)	1,5-di-O-acetyl-2,3,4,6-tetra-O-methyl mannitol	12.401
t-Gal(p)	1,5-di-O-acetyl-2,3,4,6-tetra-O-methyl galactitol	5.181
5-Ara(f)	1,4,5-tri-O-acetyl-2,3-di-O-methyl arabinitol	10.112
4-Xyl(p)	1,4,5-tri-O-acetyl-2,3-di-O-methyl xylitol	1.759
3-Man(p)	1,3,5-tri-O-acetyl-2,4,6-tri-O-methyl mannitol	1.997
2-Man(p)	1,2,5-tri-O-acetyl-3,4,6-tri-O-methyl mannitol	1.699
2-Glc(p)	1,2,5-tri-O-acetyl-3,4,6-tri-O-methyl glucitol	2.112
6-Man(p)	1,5,6-tri-O-acetyl-2,3,4-tri-O-methyl mannitol	4.180
**4-Gal(p)**	**1,4,5-tri-O-acetyl-2,3,6-tri-O-methyl galactitol**	**31.372**
**4-Glc(p)**	**1,4,5-tri-O-acetyl-2,3,6-tri-O-methyl glucitol**	**19.933**
4,6-Man(p)	1,4,5,6-tetra-O-acetyl-2,3-di-O-methyl mannitol	3.118
IPSp		
t-Ara(f)	1,4-di-O-acetyl-2,3,5-tri-O-methyl arabinitol	2.438
t-Man(p)	1,5-di-O-acetyl-2,3,4,6-tetra-O-methyl mannitol	18.763
3-Ara(f)	1,3,4-tri-O-acetyl-2,5-di-O-methyl arabinitol	1.798
t-Gal(p)	1,5-di-O-acetyl-2,3,4,6-tetra-O-methyl galactitol	7.670
5-Ara(f)	1,4,5-tri-O-acetyl-2,3-di-O-methyl arabinitol	4.935
2-Xyl(p)	1,2,5-tri-O-acetyl-3,4-di-O-methyl xylitol	2.387
3-Man(p)	1,3,5-tri-O-acetyl-2,4,6-tri-O-methyl mannitol	1.949
2-Man(p)	1,2,5-tri-O-acetyl-3,4,6-tri-O-methyl mannitol	1.064
2-Glc(p)	1,2,5-tri-O-acetyl-3,4,6-tri-O-methyl glucitol	2.267

6-Man(p)	1,5,6-tri-O-acetyl-2,3,4-tri-O-methyl mannitol	3.385
**4-Gal(p)**	**1,4,5-tri-O-acetyl-2,3,6-tri-O-methyl galactitol**	**18.997**
**4-Glc(p)**	**1,4,5-tri-O-acetyl-2,3,6-tri-O-methyl glucitol**	**27.940**
4,6-Man(p)	1,4,5,6-tetra-O-acetyl-2,3-di-O-methyl mannitol	3.958
4,6-Glc(p)	1,4,5,6-tetra-O-acetyl-2,3-di-O-methyl glucitol	2.447

Table S2 The compounds used in this work with information from NCBI PubChem compound database and the supplier sources

Compound	CAS No.	CID	MW (g/mol)	InChIKey	IUPAC name	Source
Glucose	50-99-7	107526	180.16	GZCGUPFRVQAUEE-SLPGGIOYSA-N	(*2R,3S,4R,5R*)-2,3,4,5,6-pentahydroxyhexanal	Sigma-Aldrich (St. Louis, MO, USA)
Mannose	3458-28-4	-	180.16	-	-	Sigma-Aldrich (St. Louis, MO, USA)
Galactose	26566-61-0	3037556	180.16	GZCGUPFRVQAUEE-KCDKBNATSA-N	(2*R*,3*S*,4*S*,5*R*)-2,3,4,5,6-pentahydroxyhexanal	Sigma-Aldrich (St. Louis, MO, USA)
Rhamnose	10030-85-0	20849066	182.17	CBDCDOTZPYZPRO-DEZHIRTDSA-N	(2*R*,3*R*,4*S*,5*S*)-2,3,4,5-tetrahydroxyhexanal;hydrate	Sigma-Aldrich (St. Louis, MO, USA)
Arabinose	5328-37-0	5460291	150.13	PYMYPHUHKUWMLA-VAYJURFESA-N	(2*R*,3*S*,4*S*)-2,3,4,5-tetrahydroxypentanal	Sigma-Aldrich (St. Louis, MO, USA)
Fructose	57-48-7	5984	180.16	BJHIKXHVCXFQLS-UYFOZJQFSA-N	(3*S*,4*R*,5*R*)-1,3,4,5,6-pentahydroxyhexan-2-one	Sigma-Aldrich (St. Louis, MO, USA)

Ribose	50-69-1	5311110	150.13	PYMYPHUHKUWMLA-LMVFSUKVSA-N	(2*R*,3*R*,4*R*)-2,3,4,5-tetrahydroxypentanal	Sigma-Aldrich (St. Louis, MO, USA)
						Sigma-Aldrich (St. Louis, MO, USA)
Xylose	58-86-6	135191	150.13	SRBFZHDQGSBBOR-IOVATXLUSA-N	(3*R*,4*S*,5*R*)-oxane-2,3,4,5-tetrol	Sigma-Aldrich (St. Louis, MO, USA)
Fucose	2438-80-4	3034656	164.16	PNNNRSAQSRJVSB-KCDKBNATSA-N	(2*S*,3*R*,4*R*,5*S*)-2,3,4,5-tetrahydroxyhexanal	Sigma-Aldrich (St. Louis, MO, USA)
Dextran	9004-54-0	-	1-670 kDa	-	-	International Laboratory USA (San Bruno, CA)
N-Cyclohexyl-N-[2-(4-methyl-1-oxa-4-azoniacyclohex-4-yl)ethyl]methanediimine,4-methylbenzenesulfonic acid	2491-17-0	17220	423.6	GBCAVSYHPPARHX-UHFFFAOYSA-M	-	Aladdin® (Shanghai, China)

Imidazole	288-32-4	795	68.08	RAXXELZNTBOGNW-UHFFFAOYSA-N	-	Aladdin® (Shanghai, China)
Dimethyl sulfoxide	67-68-5	679	78.14	IAZDPXIOMUYVGZ-UHFFFAOYSA-N	-	Aladdin® (Shanghai, China)
Sodium borodeuteride	15681-89-7	23673181	41.86	YOQDYZUWIQVZSF-XWFVQAFUSA-N	sodium;tetradeuterioboranuide	Aladdin® (Shanghai, China)
Dichloromethane	75-09-2	6344	84.93	YMWUJEATGCHHMB-UHFFFAOYSA-N	dichloromethane	Aladdin® (Shanghai, China)
Ammonium hydroxide	1336-21-6	14923	35.046	VHUUQVKOLVNVRT-UHFFFAOYSA-N	azanium;hydroxide	Aladdin® (Shanghai, China)
Acetic acid	64-19-7	176	60.05	QTBSBXVTEAMEQO-UHFFFAOYSA-	-	Aladdin® (Shanghai, China)
Sodium hydroxide	1310-73-2	14798	39.997	HEMHJVSKTPXQMS-UHFFFAOYSA-M	sodium;hydroxide	Aladdin® (Shanghai, China)
Acetic anhydride	108-24-7	7918	102.09	WFDIJRYMOXRFFG-UHFFFAOYSA-N	acetyl acetate	Aladdin® (Shanghai, China)
Bovine serum albumin	9048-46-8		68000	-	-	BioFroxx, (Guangzhou, China)
Acetic acid	64-19-7	176	60.05	QTBSBXVTEAM	acetic acid	Aladdin®

				EQO-UHFFFAOYSA-N		(Shanghai, China)
Propionic acid	79-09-4	1032	74.08	XBDQKXXYIPTUBI-UHFFFAOYSA-N	propanoic acid	Aladdin® (Shanghai, China)
n-Butyric acid	107-92-6	264	88.11	FERIUCNNQQJTOY-UHFFFAOYSA-N	butanoic acid	Aladdin® (Shanghai, China)
i-Butyric acid	79-31-2	6590	88.11	KQNPFQTWMSNSAP-UHFFFAOYSA-N	2-methylpropanoic acid	Aladdin® (Shanghai, China)
Valeric acid	109-52-4	7991	102.13	NQPDZGIKBAWPEJ-UHFFFAOYSA-N	pentanoic acid	Aladdin® (Shanghai, China)
KH_2PO_4	7778-77-0	516951	136.086	GNSKLFRGEWLPPA-UHFFFAOYSA-M	potassium; dihydrogen phosphate	Macklin® (Shanghai, China)
NaOH	1310-73-2	14798	39.997	HEMHJVSKTPXQMS-UHFFFAOYSA-M	sodium; hydroxide	Macklin® (Shanghai, China)
Trifluoroacetic acid	76-05-1	6422	114.02	DTQVDTLACAAQTR-UHFFFAOYSA-N	2,2,2-trifluoroacetic acid	Guangzhou (Guangzhou, China)
1-Phenyl-3-methyl-5-pyrazolone	89-25-8	4021	174.2	QELUYTUMUWHWMC-UHFFFAOYSA-N	5-methyl-2-phenyl-4*H*-pyrazol-3-one	Aladdin® (Shanghai, China)

Acetonitrile	75-05-8	6342	41.05	WEVYAHXRMPXWCK-UHFFFAOYSA-N	acetonitrile	Macklin® (Shanghai, China)
Methanol	67-56-1	887	32.042	OKKJLVBELUTLKV-UHFFFAOYSA-N	methanol	Macklin® (Shanghai, China)
Paraformaldehyde	50-00-0	712	30.026	WSFSSNUMVMOOMR-UHFFFAOYSA-N	formaldehyde	Macklin® (Shanghai, China)

CID: PubChem compound ID;

InChIKey: International Chemical Identifier

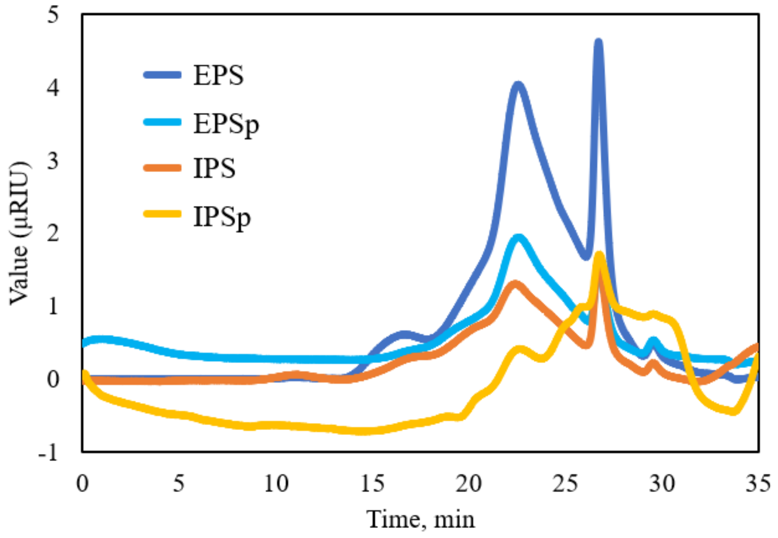

Figure S1. The gel permeation chromatography (GPC) spectra of EPS, IPS, EPSp and IPSp. EPS: exopolysaccharides, EPSp: purified exopolysaccharides, IPS: intracellular polysaccharides, IPSp: purified intracellular polysaccharides.

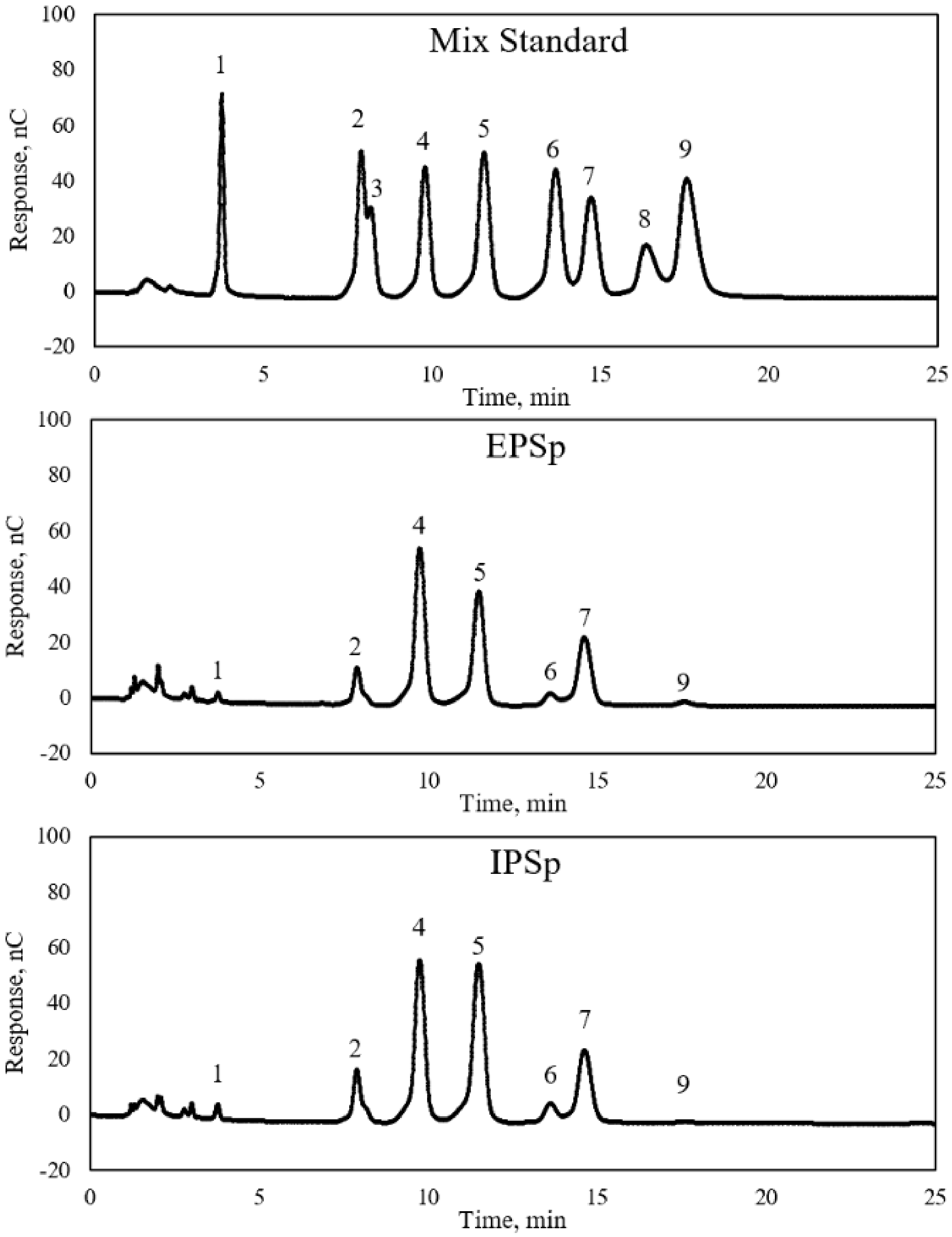

Figure S2. The ion chromatography profiles of mix standard, EPSp and IPSp. Peak (1) fucose, (2) arabinose, (3) rhamnose, (4) galactose, (5) glucose, (6) xylose, (7) mannose, (8) fructose, (9) ribose.

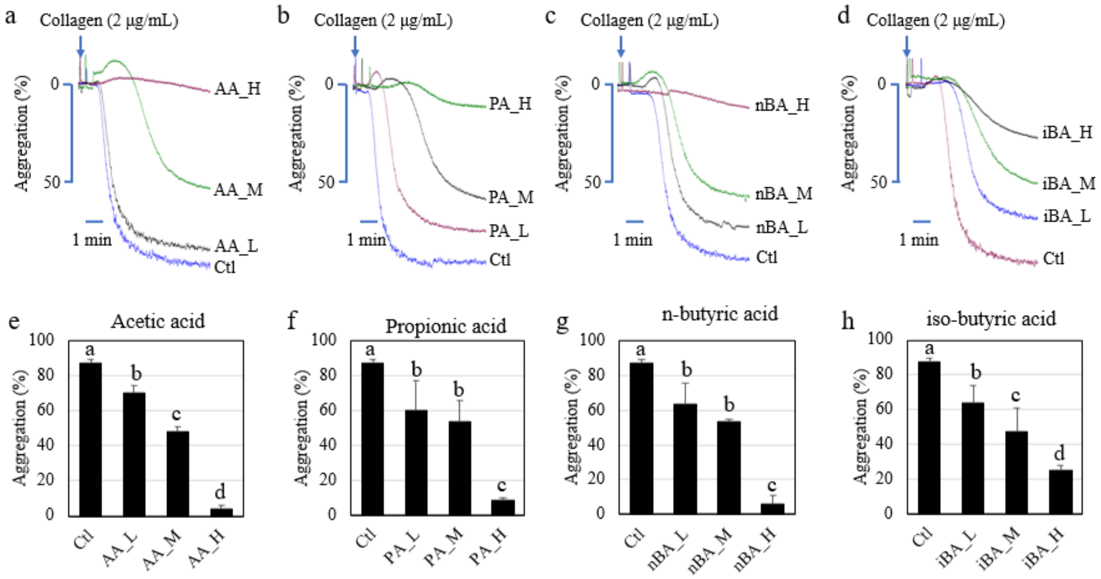

Figure S3. The effects of four individual SCFAs on human platelet aggregation induced by collagen. (a) acetic acid (AA); (b) propionic acid (PA); (c) *n*-butyric acid (nBA); (d) *i*-butyric acid (iBA); (e-h): bar charts of individual SCFAs. L: 20 μg/mL, M: 40 μg/mL, H: 60 μg/mL. Collagen: 2 μg/mL. Gel-filtered platelets (2.5 × 10^8 cells per mL) were pre-incubated with individual SCFAs for 40 min at 37 °C. Data are presented as mean ± standard deviation, n = 3. Different letters indicate significant difference among different groups for the same SCFA, $p < 0.05$, ANOVA.

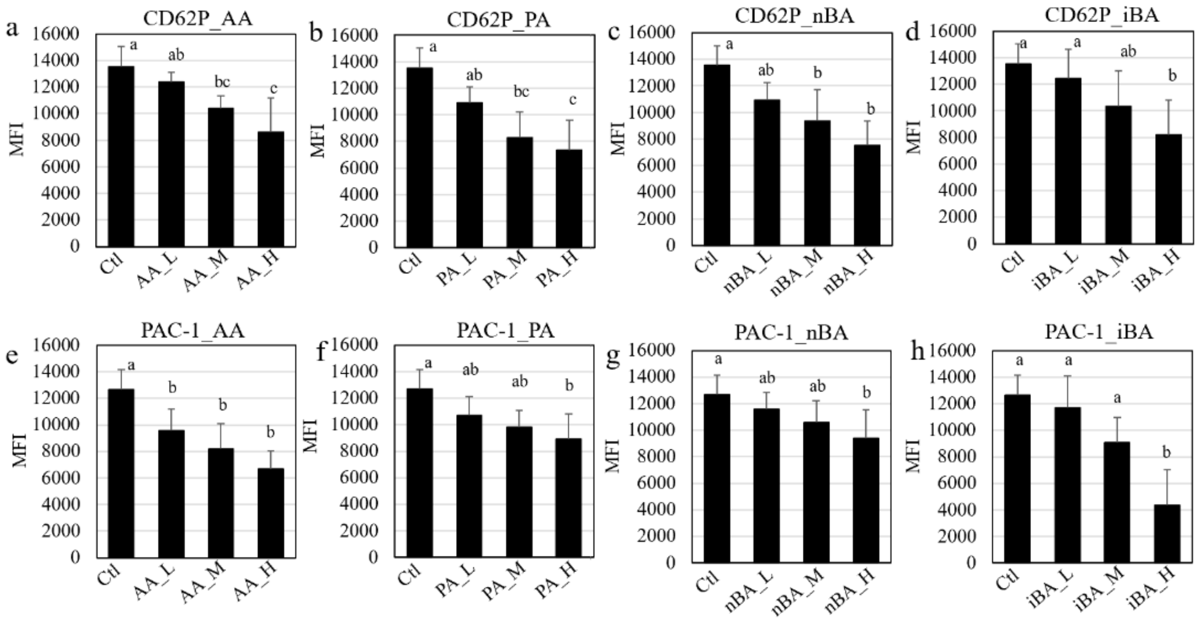

Figure S4. The effects of four individual SCFAs on human platelet activation. (a-d) CD62P expression; (e-h) PAC-1 binding to platelets. AA: acetic acid; PA: propionic acid; nBA: *n*-butyric acid; iBA: *i*-butyric acid. L: 20 μg/mL, M: 40 μg/mL, H: 60 μg/mL. Collagen: 2 μg/mL. Data are presented as mean ± standard deviation, n = 3. Different letters indicate significant difference among different groups for the same SCFA, $p < 0.05$, ANOVA.

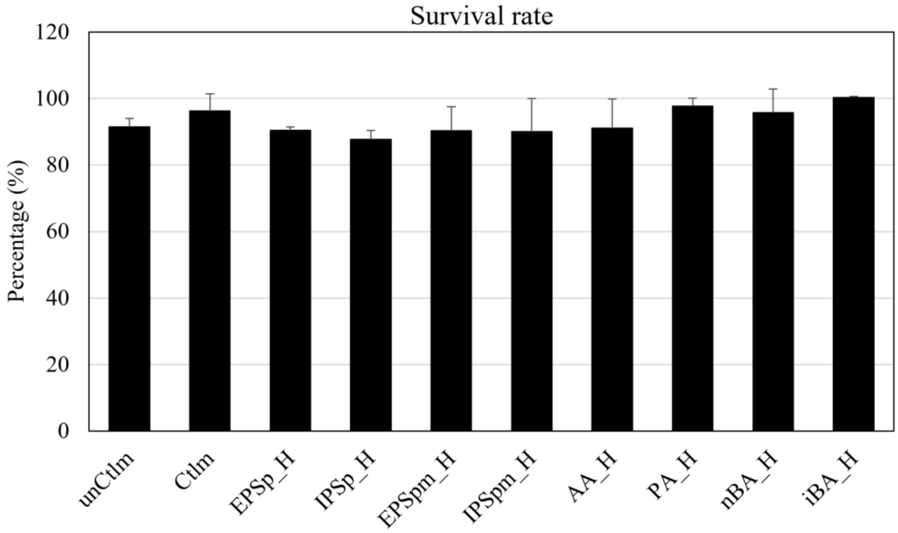

Figure S5. The effects of EPSp, IPSp, their bacterial metabolites and SCFAs on the viability of platelets. The survival rate of Ctl was normalized to 100%. Ctl: phosphate buffered saline; Unctlm: fecal fermentation basal medium before fecal fermentation; Ctlm: fecal fermentation basal medium after fecal fermentation; EPSp: purified Cs-4 exopolysaccharides; IPSp: purified Cs-4 intracellular polysaccharides; EPSpm: EPSp after fecal fermentation; IPSpm: IPSp after fecal fermentation; AA: acetic acid; PA: propionic acid; nBA: *n*-butyric acid; iBA: *i*-butyric acid. H: 5 mg/mL for EPSp, IPSp, EPSpm and IPSpm, and 60 μg/mL for SCFAs. Data are presented as mean ± standard deviation, n = 3. $p < 0.05$, ANOVA.

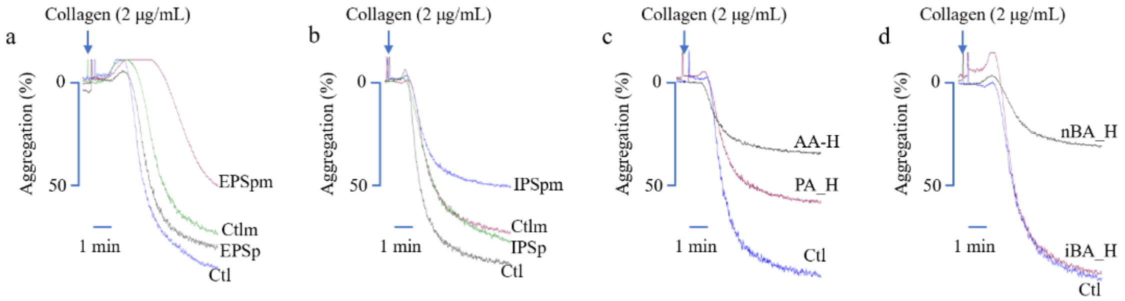

Figure S6. The anti-aggregation effects of EPSp, IPSp and their bacterial metabolites on platelet rich plasma (PRP). (a) EPSp and EPSpm; (b) IPSp and IPSpm; (c) acetic acid (AA) and propionic acid (PA); (d) *n*-butyric acid (nBA) and *i*-butyric acid (iBA). Ctl: phosphate buffered salin; Unctlm: fecal fermentation basal medium before fecal fermentation; Ctlm: fecal fermentation basal medium after fecal fermentation; EPSp: purified exopolysaccharides, IPSp: purified intracellular polysaccharides. EPSpm: EPSp after fecal fermentation; IPSpm: IPSp after fecal fermentation. PRP (2.5×10^8 cells per mL) was pre-incubated with EPSp, IPSp, EPSpm and IPSpm (H: 1 mg/mL, 4 : 1, v/v) or different controls (Ctl, Unctlm and Ctlm, 1 : 4, v/v) for 40 min at 37 °C. Collagen: 2 μg/mL.

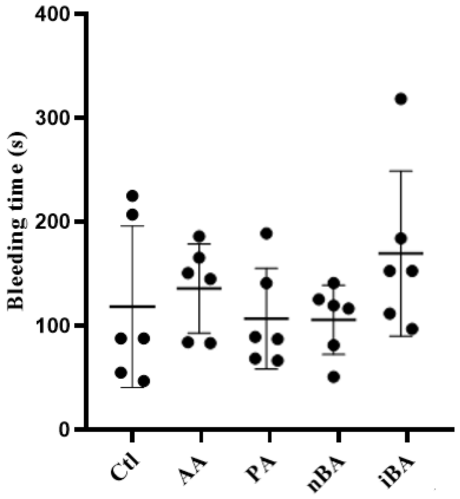

Figure S7. The effects of SCFAs on tail bleeding time in C57BL/6J mice. Ctl: phosphate buffered salin; AA: acetic acid; PA: propionic acid; nBA: *n*-butyric acid; iBA: *i*-butyric acid. Each black dot represents a mouse. Data are presented as mean ± standard deviation, n = 6. $p < 0.05$, ANOVA.

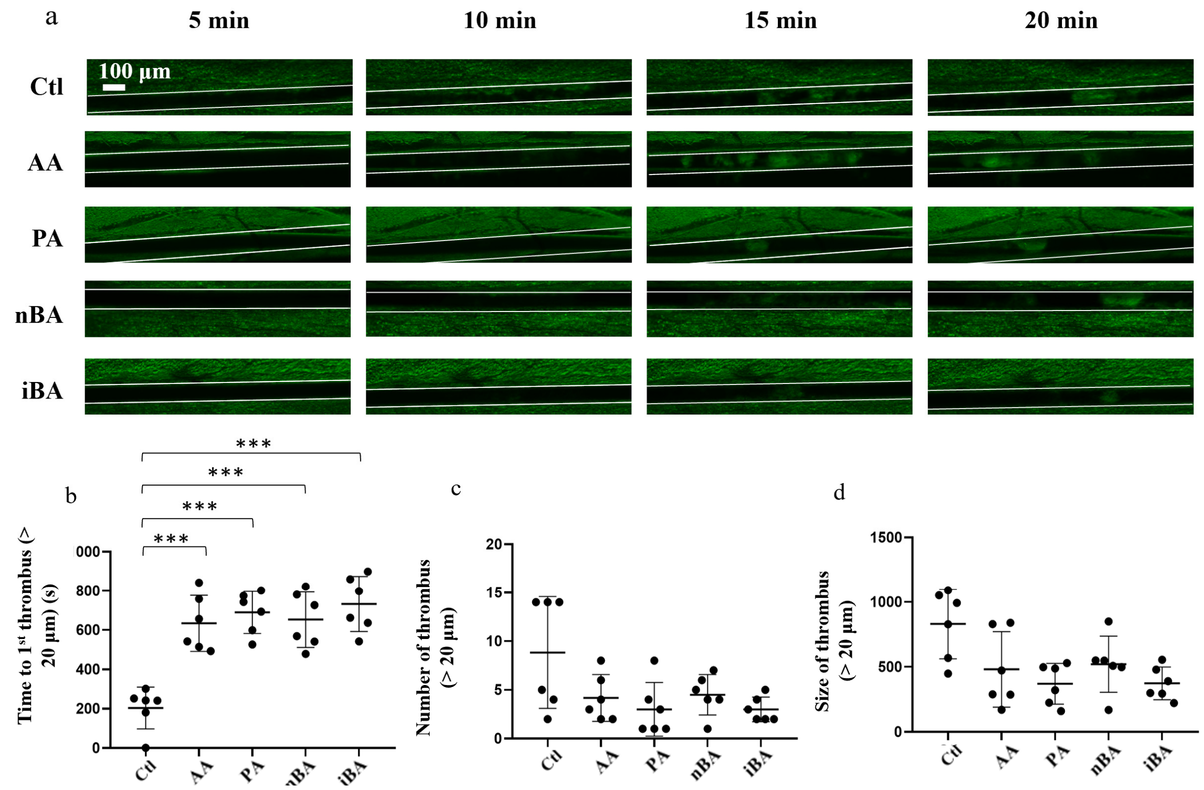

Figure S8. The effects of SCFAs on thrombus formation induced by $FeCl_3$ in C57BL/6J mice. (a): presentative pictures of mesenteric arterioles at different time points; (b): time to 1st thrombus (> 20 µm); (c) number of thrombus (> 20 µm); (d) size of thrombus over 2 min starting from the time of the first thrombus (> 20 µm). Ctl: phosphate buffered salin; AA: acetic acid; PA: propionic acid; nBA: *n*-butyric acid; iBA: *i*-butyric acid. Data are presented as mean ± standard deviation, n = 6. *** indicates significant difference among different groups for the same SCFA, $p < 0.001$, student's *t*-test.
